# ATTITUDES TOWARD OWN AGING AND PERSONALITY IN LATER LIFE: EXAMINATION OF BIDIRECTIONALITY OVER 20 YEARS

**DOI:** 10.1093/geroni/igz038.1413

**Published:** 2019-11-08

**Authors:** Anna E Kornadt, Jelena S Siebert, Hans-Werner Wahl

**Affiliations:** 1 Bielefeld University, Bielefeld, Nordrhein-Westfalen, Germany; 2 Heidelberg University, Heidelberg, Baden-Wurttemberg, Germany

## Abstract

Big Five personality traits are assumed to be linked with attitudes toward own aging (ATOA). Both constructs have central importance for the aging process, it is thus important to comprehensively address their mutual connection over time. We used data from the ILSE study, a longitudinal study with four measurement occasions, spanning 20 years and including two participant cohorts (n = 501; born 1950-52 and n = 500; born 1930-32). Dual latent change score models showed that personality was longitudinally related to change in ATOA: Lower Neuroticism, higher Conscientiousness, and higher Openness predicted more positive attitudes; the effect for Extraversion varied by time. Furthermore, the role of personality seems to be confined to certain sensitive periods in midlife and early old age. ATOA had only marginal longitudinal impact on personality. Our results shed light on the developmental co-dynamics of personality and subjective perceptions of aging across the second half of life.

